# Thinking about Kindergarten thinking: A mixed methods study

**DOI:** 10.3389/fpsyg.2022.933541

**Published:** 2022-09-02

**Authors:** Heather Braund

**Affiliations:** Faculty of Education, Queen’s University, Kingston, ON, Canada

**Keywords:** metacognition, teacher, early years, self-regulation, Kindergarten

## Abstract

Metacognition, otherwise known as ‘thinking about one’s thinking,’ leads to greater academic success and is foundational. Given this importance, metacognitive behaviors need to be developed within early years contexts to provide young children the opportunity to practice these behaviors and receive feedback. However, literature continues to focus on the development of metacognition in later grades. This mixed methods study explored metacognition in eight Kindergarten classrooms. Participants included eight Kindergarten teachers, six early childhood educators (ECEs), and 80 students. Data collection was conducted at two time periods separated by 12 weeks. Data collection included the Children’s Independent Learning Development (CHILD) measure, semi-structured interviews, and classroom observations. The quantitative data from the CHILD were analyzed using a paired samples *t*-test in SPSS. All qualitative data were analyzed thematically. Qualitatively, three themes were identified: (1) Conceptualization of metacognition, (2) Barriers to developing metacognition, and (3) Operationalization of strategies to facilitate metacognitive development. Evidence demonstrated that participants had incomplete conceptualizations of metacognition. Some articulated simple understandings by reporting the literal translation and were unable to articulate more fulsome conceptions. However, some teachers had more developed conceptions of metacognition that included different facets such as planning and reflective thinking. All participants were forthcoming with identifying ways in which they struggled with implementing metacognitive practices or encouraging the development of metacognition. These barriers included large classroom sizes, developmental readiness, and wide student ability. Despite having an incomplete understanding of metacognition, early years educators were trying a variety of different strategies to help promote metacognitive thinking within their Kindergarten classrooms. Surprisingly, teacher ratings using the CHILD did not change significantly from Time 1 (*M* = 1.88, SD = 0.744) to Time 2 (*M* = 1.85, SD = 0.66), *t* (72) = 0.72, *p* > 0.05. A similar trend was observed for ECE ratings using the CHILD as they did not change significantly from Time 1 (*M* = 1.89, SD = 0.70) to Time 2 (*M* = 1.80, SD = 0.79), *t* (52) = 1.36, *p* > 0.05. This research highlights empirical practices that Kindergarten educators can use to help facilitate metacognitive thinking. Furthermore, it identifies a need to better support Kindergarten educators by integrating practices aimed at developing metacognitive thinking in their students through explicit examples of strategies.

## Introduction

Scholars widely accept that the ability to be metacognitive and self-regulate is crucial for successful learning within the classroom (Perry et al., 2018) and beyond (Boekaerts and Cascallar, 2006). Furthermore, research suggests that components of metacognition and self-regulation (SR) begin to develop in young children. Hence, there is a need to support and further develop these skills throughout elementary school contexts and beyond. The following introduction will describe components of metacognition, discuss the relationship between metacognition and other regulatory behaviors, outline its development in the early years, and identify key facilitators impacting the development of metacognition.

### Conceptualizing metacognition

Metacognition as a construct has evolved over the years from when it was originally defined as ‘Thinking about thinking’ by Flavell (1979). Furthermore, metacognition was originally believed to be comprised of conscious actions (Flavell, 1979). There were two main components of metacognition known as metacognitive knowledge (MK) and metacognitive regulation. The component of MK includes the beliefs and thoughts that an individual has about their own or another individual’s cognitive processes (Flavell, 1979). Metacognitive regulation [also known as metacognitive skills (MS)] is a more active component that includes the process of monitoring, controlling, and evaluating learning outcomes (Efklides, 2006). A third component of metacognition is known as metacognitive experiences (MEs) which encompasses the judgments and feelings that individuals have about learning (Efklides, 2006; Ben-David and Orion, 2013).

These conceptualizations have expanded to include more social interactions that may help to facilitate the development of metacognition. For example, scholars acknowledge that metacognition is not necessarily an individual phenomenon and may be shaped by social interactions (Moraitou and Metallidou, 2021). Additionally, there is ongoing intrigue regarding the interaction between metacognition and affect across the life span (Moraitou and Metallidou, 2021). Specific to the level of consciousness required, Efklides proposed a model of metacognition which suggests that aspects of metacognition may occur at a non-conscious level (Efklides, 2008).

### Conceptual framework

This study was guided by the model of metacognition as described by Efklides (2008). The multifaceted and multi-level model consists of three levels (object level, metalevel, and meta-metalevel) with opportunities for monitoring, reflection, and control. The object level includes processes specific to cognition and emotion that occur at a non-conscious level. Monitoring and control are two non-conscious regulatory systems involved in developing products at the metalevel. These products are elements of self-awareness such as emotions and thoughts alongside ME, MK, and MS. This is the level where thoughts and interpretations of the learning situation become conscious. Similarly, both control and regulatory systems are actively involved at the metalevel. More specifically, when control is required, MEs and MK may activate MS. Metacognitive feelings (a component of ME) play an important role whereby they can activate the regulatory loop as necessary. The final level, the social level, only includes metacognitive judgments which may be about the individual or others’ metacognition capacity (ME, MK, and MS). This level is also informed by the personal-awareness level and by interactions with others. The monitoring processes at this level are conscious and may be in the form of reflecting. Similarly, the control process is also conscious (Efklides, 2008).

### Relationship between metacognition, self-regulation, and self-regulated learning

Metacognition, SR, and self-regulated learning (SRL) have been closely intertwined for years. Some scholars have even used the terms interchangeably given the role of monitoring and regulatory processes (Dinsmore et al., 2008). However, conceptually, developmentally, and about measurement, some scholars have identified differences between the three constructs. Yet, the debate continues as to which construct emerged first and whether there is an overarching construct (Veenman et al., 2006; Gascoine et al., 2017). Given the complexity of these constructs and the lack of conceptual clarity around nesting within constructs, a hierarchical approach to studying metacognition is not appropriate (Gascoine et al., 2017).

A self-regulated individual can control their thoughts, emotions, and behaviors as they work toward attaining their goals (Zimmerman, 2000; McClelland and Cameron, 2012). SRL is a sub-component of SR whereby the cognitive, social, and behavioral processes are focused entirely on an individual’s learning (Dinsmore et al., 2008). We know that metacognition is necessary to engage in successful SRL (Boekaerts, 1999). Furthermore, MEs are particularly useful as evidenced in the model proposed by Efklides whereby ME can trigger the regulatory loop while learning.

### Measurement of metacognition in early years

Earlier scholars believed that metacognition developed in older students typically between the ages of 8 and 10 (Veenman et al., 2006). However, scholars have since challenged this understanding by examining SR development in younger children (Bronson and Bronson, 2001). There has now been a keen interest in understanding the development of both metacognition and SR in the early years (Blair and Razza, 2007; Dignath et al., 2008; Whitebread et al., 2010; Erdmann and Hertel, 2019; Perry, 2019). Alongside the interest in exploring metacognition in young children, comes an emphasis on using more developmentally appropriate methods for measuring metacognition (Perry, 2019). Earlier research used self-report methods (Winne and Perry, 2000) which were problematic given the reliance on accurate reporting of cognitive processes by respondents. However, this was even more problematic when used with young children who may have been incapable of reporting or verbalizing their thoughts and skills related to metacognition. Therefore, other methods of collecting data including observing the behaviors directly were recommended (Winne and Perry, 2000; Whitebread et al., 2010). One systematic review examined how metacognition was assessed in children between the ages of 4 and 16 years and demonstrated that self-report measures were used by 61% of the studies included in the review but were only used with children of ages 7 and older (Gascoine et al., 2017). Observational methods were used with children between the ages of 4 and 8 including think aloud protocol whereby the individual is prompted to explain their thinking verbally while completing an activity. Teacher ratings were used with children aged 4 and above whereas task-based methods were only used with children aged 7 and older (Gascoine et al., 2017). This review highlights that teacher ratings and observational methods have been used with young children. To help understand the complex phenomenon of metacognition, a multi-method design has been recommended including observations of student behavior (Veenman, 2005), which can then be triangulated with other measures.

### Developing metacognition in the early years

Given that metacognition remains blurred conceptually and in practice with other concepts closely intertwined such as SR and SRL, there is no standard set of practices agreed upon by scholars that can be used to promote the development of metacognition (Perry et al., 2018). Although given our conceptual understanding, we could expect educators would use strategies related to planning, evaluating, and regulating an individual’s performance (Perry et al., 2018). Sometimes the strategies may be focused on a specific domain such as mathematics (Dignath et al., 2008) or science (Zohar and Barzilai, 2013). Researchers have also demonstrated the value of developing metacognition across the curriculum (Perry et al., 2012). Some recommendations for facilitating the development of metacognition include that it should be embedded throughout lessons rather than teaching it through disconnected or singular lessons, the purpose including the focus on metacognition should be made explicit to the learners, and the learning should be longitudinal (Veenman et al., 2004). Another suggested practice includes the integration of group work (Perry et al., 2018), which encourages learning with and from others.

There are a variety of factors that have been identified as facilitating the development of metacognition such as the role of formative assessment. Assessment for Learning *(AfL)* is a component of formative assessment with increased student agency. *AfL* is the process of collecting data about student learning with the ultimate goal of co-constructing these practices (Adie et al., 2018) and improving student learning (Group, 2002). Assessment as Learning *(AaL)* practices, described as a subcomponent of AfL, includes the process by which students reflect and evaluate their learning to enhance their metacognition and SRL development (Earl, 2013). The relationship between metacognition and *AfL* was explored empirically in 528 students ranging from grade four to six across seven Dutch elementary schools. Findings highlighted that monitoring strategies predicted planning activities. Monitoring and planning also had an effect size of 0.26 with scaffolding practices ranging between 0.25 and 0.36 in their effect sizes. Furthermore, scaffolding practices were positively correlated with the use of learning strategies and the evaluation of their learning. Finally, the use of metacognitive strategies facilitated the use of AfL strategies (Baas et al., 2015). Another study conducted in Ontario, Canada explored the connections between assessment and metacognition through five purposefully selected interviews with elementary teachers. One key theme highlighted how teachers modified their practices to support the development of students’ metacognition. A two-way feedback process helped teachers to modify their practices as they often sought feedback from their students on how they could further support them. *AaL* practices were also described as essential when developing metacognition. This included assessment practices such as success criteria, descriptive and ongoing feedback, peer-assessment, self-assessment, conferencing, portfolio use, and reflective thinking activities for use to understand student thinking. All participants reported needing additional support to help them with increasing student agency and developing metacognition through assessment (Braund and DeLuca, 2018). These two studies provide examples of a direct empirical connection between metacognition and formative assessment.

However, metacognitive skills at a young age need to be developed in collaboration with other individuals such as teachers who are well positioned to provide explicit instruction and modeling for how to use metacognitive strategies. A common method for encouraging students to think metacognitively is through the use of prompting questions. For example, Jacobs (2004) explored the metacognitive awareness of Kindergarten students through the writing process. The students would observe the teacher doing a think-aloud about different elements of the writing process and then were given time to work on their writing. The teacher worked with the students during the writing period and then peers provided feedback after the dedicated writing period. The researcher would then interview students to ask them questions prompting them to reflect on their thinking and writing. There were some answers to questions that students did not know originally but this changed over the course of the year. By the end of the year, all students demonstrated that they were capable of explaining their thinking using metacognitive terminologies such as “thought” or “mind” and were also able to provide examples of strategies that they had used during their writing time (Jacobs, 2004). This study is one example demonstrating the potential for students to develop their metacognitive strategy use and metacognitive regulation over time and as young as the Kindergarten level. Although the Jacobs study was specific to literacy, many of the prompting questions could be adapted for use in other subject domains such as science or math where ‘thinking aloud’ can be very beneficial for the development of metacognition in young children as a means for making educator thinking visible. This study also reiterates the important role that educators play in the development of metacognition, especially for young children at the Kindergarten grade level.

A more recent study conducted by Dörr and Perels (2019) examined the effectiveness of an intervention designed to improve metacognitive skills in 137 children in Kindergarten. Teachers and parents received training on specific strategies that they could use in the classroom (for teachers) and in the home environment (for caregivers) to develop metacognition in their children. The children then were filmed while completing a problem-solving task which was later coded for metacognitive behaviors by two observers. The categories for coding behaviors related to monitoring, control, and lack of monitoring and control. Their findings highlighted that students were able to demonstrate an improvement in the control aspect of metacognition; however, monitoring seemed to be more challenging. However, the authors acknowledge that it is easier to observe control strategies than monitoring strategies which may act as a limitation (Venitz and Perels, 2019). Despite the importance of developing these skills in the early years, much of the research continues to focus on metacognition at later stages of development such as in secondary and post-secondary contexts. Therefore, this mixed methods study explored the development of metacognition in Kindergarten classrooms and was guided by the following questions: (1) How do early childhood educators (ECEs) conceptualize and articulate metacognition? and (2) How do young children’s metacognition and self-regulatory behaviors evolve across two time periods as measured by educators?

## Materials and methods

This concurrent mixed methods (Creswell and Plano Clark, 2011) study explored metacognition development in Kindergarten. Given the complexity of metacognition and the evolving understanding in the early years, the research design required a complex intersection of data sources (Plano Clark and Ivankova, 2016). The quantitative data described the evolution of metacognition behaviors, whereas the qualitative data provided some context for why these behaviors may occur and how they are developed. It is important to note that these data were collected as part of a larger dissertation study.

### Context

This study was conducted in Ontario, Canada where a play-based approach is mandated across Kindergarten classrooms in the public education system. Kindergarten students attend school daily and are supported by a teaching team that includes one Kindergarten teacher and one registered ECE (OME, 2016).

### Participants

A total of eight Kindergarten teachers and six ECEs agreed to participate in this study during the 2018–2019 year. All teachers and ECEs self-selected to participate and indicated that they had an interest in SR. Demographic information for the teachers and ECEs has been previously reported (Braund et al., 2021). Teachers and ECEs were recruited from one Ontario school board, across five publicly funded elementary schools. Despite recruiting from only one school board, the participating schools were purposefully diverse with some located in the city and one located rurally. Additionally, the schools were sampled across socioeconomic status (SES) with some schools in higher SES areas, a school located in a moderate SES area, and one school located in a low SES area. A snowball sampling technique was used where the recruitment message was circulated to the early years and assessment coordinators within the school board who passed along the information to principals and teachers. To help increase the likelihood of representation, teachers and ECEs were diverse and had a range of practice experiences. All teachers and ECEs provided informed written consent. Once they had consented, the teacher from each participating class shared the Letter of Information with the parents of students in their classes. Parents were made aware that having their children participate was voluntary and were asked to provide written consent for their child to participate. Additionally, oral assent was also collected from each student before any tasks were administered. Participants were able to withdraw from the study up until July 2019 after which withdrawal was no longer possible. None of the participants withdrew from the study. A total of 80 students were recruited to participate across the eight classrooms. However, due to attrition, only 77 students participated across both time periods. There was a fairly even split across the data with 39 female students and 41 male students. A similar breakdown was observed according to grades with 40 students in Junior Kindergarten and 40 students in Senior Kindergarten (SK).

### Data collection procedures

There were two time periods for data collection. Time 1 was in the winter of 2019, whereas Time 2 was in the spring of 2019. There were approximately 12 weeks in-between Time 1 and Time 2 to try and facilitate the development of student metacognition and SR. The researcher spent an orientation day in each of the eight classrooms to learn more about the context and familiarize herself with the students, teacher, and ECE. Following the orientation day, a total of 3 days were spent in each classroom during each time period. This amounted to 448 h spent collecting data across the eight classrooms. Additional data sources were collected as part of the larger dissertation study that is beyond the scope of this paper. The independent semi-structured interviews with teachers and ECEs were conducted on the 3rd day in each classroom for each time period. A total of 16 interviews were conducted with teachers and eight with ECEs. Two of the ECEs opted to not participate in the semi-structured interviews. The Children’s Independent Learning Development (CHILD) measure was given ahead of time to teachers and ECEs. They were asked to complete it for each participating student in preparation for each time period.

The researcher hired six research assistants (RAs) to help with data collection. All RAs were trained in how to observe teachers, ECEs, and students. A few were also trained in how to conduct semi-structured interviews. However, most interviews were conducted by the researcher except for when there were scheduling conflicts.

### Measures

Three measures were used to collect data for this study: semi-structured interviews, classroom observations, and observations collected using the Children’s Independent Learning Development (CHILD) measure (Whitebread et al., 2009). The semi-structured interviews had protocols specific to each time period. At Time 1, the focus of the interviews was on understanding conceptions of classroom assessment, SR, and the relationship between these constructs. For example, educators were asked to describe what a student who is able to self-regulated looks like in their classroom and to discuss the relationship (if any) between formative assessment and SR. Additionally, educators were also asked to identify any challenges impacting their ability to promote SR and recommend any resources or supports that would help them with developing SR. The interview at Time 2 was more focused on identifying examples of assessment practices and efforts to develop SR. Educators were asked to answer a number of questions including sharing their understanding of metacognition and examples of how it was operationalized in their classrooms. They were also asked about the use of self-assessment and to share examples of how they had integrated the assessment practice. The interview protocols were developed by a team of assessment and SR experts and then piloted before use in the current study. This helps to enhance the trustworthiness of the data collected and inferences made as a result of the interview protocols. The interview protocols and additional details on piloting have been published previously (Braund et al., 2021). The interviews were conducted in either the staff room or in the classroom when students and others were not present. All interviews were audio-recorded and transcribed verbatim. The interviews lasted on average 41 min for Time 1 and 24 min for Time 2.

The classroom observations did not follow a structured protocol given that many of the other measures used in the larger study were very structured. The observation periods were used as an opportunity to capture important contextual information and concrete practices used to develop metacognition and SR. More specifically, the observations were used to document field notes that related to assessment and SR practices as demonstrated by educators (teacher or ECE) or students. Although it is important to note that only classroom observations pertaining to metacognition are presented in this paper. Therefore, for teachers, it captured example practices of ways in which they were encouraging the students to self-regulate. An example related to metacognition was observing and documenting the use of a think-aloud protocol in math by one Kindergarten teacher. The mechanism for identifying changes in behavior was by comparing observations at Time 1 and Time 2. In addition to the field notes, direct quotations were captured when possible. All RAs shadowed the researcher for at least one day in a classroom to understand how to observe students, teachers, and ECEs. During this shadowing process, the RAs and researcher would document classroom observations independently and then compare their notes. The researcher would provide the RA with additional feedback and guidance as necessary. This modeling and scaffolding process helped to increase the trustworthiness of the data collected and inferences made as a result of the classroom observations.

The CHILD measure (Whitebread et al., 2009) is an observation protocol that was used to record students’ self-regulatory behaviors. This measure consists of 35 statements informed by metacognition and SR literature. It was previously validated by the developers and demonstrated an ability to discriminate between three levels (high, intermediate, and low) of metacognition/SR/independence. However, they continued with further piloting and finalized a 22-item shorter protocol. These items were grouped across four areas of SR: emotional (five items), prosocial (five items), cognitive (seven items), and motivational (five items). The 22 items were pilot tested a total of 576 times across 192 children. Their reliability analysis identified a high level of internal consistency (Cronbach’s alpha = 0.97). The authors outlined three ways that they addressed issues of validity when developing the CHILD including collecting the data within the classroom context or the natural environment, involving teachers in the analysis process given their expertise in classroom contexts, and recording the data for additional analysis (Whitebread et al., 2009).

This protocol was modified by the researcher to help the current participants with interpreting the items. The modification consisted of adding numbers to the frequency categories. Therefore, teachers and ECEs were asked to report the frequency of students’ self-regulatory behaviors as always (3), usually (2), sometimes (1), or never (0). In addition to providing the frequency for each item, participants were also provided with the space to add comments related to each item. The narrative comments were optional. They sometimes provided additional context for why the educators had rated that level of frequency for the item. As an additional reliability and validity measure, the researcher met with each teacher and ECE to discuss the measure before they completed the protocol. More specifically, the researcher discussed each item in detail and explained that the protocol was an overview of what they had observed to date for each item. This measure was entirely completed by the teacher and ECE for each participating student.

### Data analysis procedures

#### Quantitative

All quantitative data were entered into an Excel spreadsheet once data collection had finished. All descriptive and inferential statistics were performed using the Statistical Package for the Social Sciences (SPSS, Version 27). When there were instances of missing data, the quantitative analyses were run listwise. The level of significance was set at .05 for all quantitative analyses. The researcher checked for internal consistency every time the CHILD measure was used and values suggesting a high level of internal consistency were found across participants including for ECEs at Time 1 (Cronbach’s alpha = 0.97,) ECEs at Time 2 (Cronbach’s alpha = 0.97,) Teachers at Time 1 (Cronbach’s alpha = 0.98) and Teachers at Time 2 (Cronbach’s alpha = 0.97). These high levels of internal consistency help to provide validity evidence specific to the internal structure of the 22 items grouped together for the CHILD measure. For the Children’s Independent Learning Development (CHILD) measure, a total score was calculated for each time period. The highest score per item was 3 and there was a total of 22 items. Therefore, the maximum possible total score that a student could have received was 66. A total score was calculated for the teacher ratings per child at each time period. This variable was calculated again for the ECE ratings per child at each time period. A mean score was computed for each child at Time 1 and again at Time 2. The mean score was calculated for the teacher ratings and then again for the ECE ratings. A paired *t-*test was used to identify changes in students’ scores on the CHILD across time periods for teacher ratings. A paired *t*-test was also used to identify changes in students’ scores on the CHILD across time periods for ECE ratings. Data were analyzed using a listwise approach. Therefore, due to missing data (e.g., students moving away), a total of 73 students were included in the quantitative dataset for teachers and only 53 for ECEs. It is important to remember that only four of the possible eight ECEs participated hence the lower number of students. Demographics were reported according to sex (male and female) and grade level (junior kindergarten and SK).

#### Qualitative

All transcripts and classroom observations were uploaded into NVivo (Version 12) for analysis. Data were analyzed using an inductive thematic approach (Braun and Clarke, 2006). All transcripts and classroom observations were read in full before the coding process commenced. To enhance trustworthiness, a second researcher coded 20% of the data independently which was then compared with the primary researcher. This process included a selection of diverse transcripts which once coded were compared line by line. An inter-coder reliability level of 94% was calculated by documenting the number of times that the two researchers agreed on each line of coding divided by the number of times the two researchers disagreed and then multiplied by 100 to obtain the percentage. This dialog and reflexive process resulted in a consensus-built codebook. Given the high level of agreement, it seemed appropriate for the primary research to complete the remainder of the coding using the consensus-built codebook (Cofie et al., 2022). Three levels of coding were performed. The smallest unit of analysis was a code. For the first level of coding, each transcript and affiliated classroom observations were coded individually. This process of open coding resulted in the assignment of a code to each segment of text. After open coding was complete across all transcripts and classroom observations, all documents were analyzed again with a focus on creating subthemes. To identify subthemes, similar codes were grouped together. The final level of selective coding consisted of reviewing all qualitative data with a focus on grouping similar subthemes together to form broad themes across the data. The researcher maintained an audit trail of any new codes that were added, any codes that were renamed, and any codes that were merged with the rationale for every change made. Thematic saturation was reached after analyzing five of the eight teachers and after the third ECE.

### Trustworthiness and researcher reflexivity

The researcher made ongoing efforts to increase trustworthiness throughout the research process. The four criteria of rigor guided these efforts including credibility, transferability, dependability, and confirmability (Guba, 1981). The first criterion, credibility suggests that the results are true, credible, and believable. Some strategies for this include prolonged engagement in the setting. This was achieved by spending 448 h across the classrooms observing and interviewing participants. Piloting the interview protocol and the CHILD measure as described earlier also helped to enhance the credibility. Dependability is centered around the extent to which the study could be replicated. The rich description of the study methods aids with replicability. Additionally, inter-coder reliability processes can enhance dependability as described earlier following recommended guidelines (Cofie et al., 2022). Confirmability is the extent to which other researchers could confirm the findings. Maintaining a reflexive process contributes to this rigor. The researcher maintained a research journal throughout the entire research process right from idea conception through to dissemination. This journal was used to identify common patterns, unique findings, document questions for discussion with her dissertation committee, note possible biases, and make fieldnotes from the interviews and classroom observations. The process of triangulating across measures also contributes to confirmability. Therefore, two measures were used to collect this data. The classroom observations were used to confirm what educators reported in their interviews but also to capture practices not described in the interviews. All participants were also offered the opportunity to review key findings from their transcripts as a form of member-checking but only one teacher acknowledged the findings and confirmed the interpretation. Finally, transferability refers to the extent to which the results could be applied to other contexts. Purposeful sampling aids with transferability and was thus used in this study. All educators were interested in SR, therefore, increasing the likelihood that they would integrate practices aimed at developing student metacognition.

## Results

The findings are organized below according to the two research questions. For the qualitative data (classroom observations and interviews), sample quotations have been provided in-text. However, additional quotations are available in Supplementary Appendix A. ECE will represent quotations from ECEs. Finally, classroom observations will be clearly outlined in brackets following the direct example.

### Research question (1) how do early childhood educators conceptualize and articulate metacognition?

Qualitatively, a total of three themes were identified from the classroom observations and interviews with early educators: (1) Conceptualization of metacognition, (2) Barriers to developing metacognition, and (3) Operationalization of strategies to facilitate metacognitive development.

#### Theme 1: Conceptualization of metacognition

Findings demonstrated that both teachers and ECEs had incomplete conceptualizations of metacognition. Some articulated simple understandings by reporting the literal translation and were unable to articulate more fulsome conceptions. For example, this teacher simply said, *“thinking about thinking”* (Teacher 4, Time 2) when she was asked to describe metacognition. One teacher asked for the definition of metacognition indicating a lack of understanding. Another teacher mentioned one component *“Understanding your thinking”* (Teacher 5, Time 2) highlighting more than just the literal translation of the construct. However, a small sample of educators had more developed conceptions of metacognition that suggested metacognition was multi-faceted and included multiple components such as planning and reflective thinking. One teacher shared a resource that she used relating to metacognition:

I love the book, “Pedagogical Documentation in Early Childhood.” This book relates to metacognition and the importance of reflection of observations made in tracking student success and behaviors (Teacher 3, Time 2).

One of the most discussed facets of metacognition was awareness. Sometimes the awareness was in relation to *“the prior knowledge they need to possess”* as described by ECE2 at Time 2. Educators also described the importance of having an awareness of how you process information. Multiple educators also mentioned awareness of thinking as described by this educator *“being aware of your thought processes”* (ECE4, Time 2). A different educator shared how metacognition related to other constructs as explained here *“I feel it is important for the upper levels of Maslow’s [Hierarchy of Needs] including a strong growth mindset and connections to mental health and resiliency*” (Teacher 7, Time 2). Discussing metacognition beyond the literal translation and identifying key components suggested a deeper understanding of the construct. The last subtheme centered around student capacity. Most educators in this study reported that Kindergarten students were able to be metacognitive. For example, this teacher explained further “I think they’re much more capable than you would maybe imagine” (Teacher 3, Time 1). However, one teacher readily identified the sophistication of the skill below:

Developmental readiness to a degree because I feel…even as an adult when I think about myself for me to have reflective practice and to think about “What am I doing? What can I be better at as a teacher? What can I be better at as a learner?” That’s a pretty sophisticated, complex skill (Teacher 1, Time 1).

Despite the complexity of metacognition, the same teacher was able to identify students in her classroom who were capable and *“I’ve had kids who in the past who have been really good at that. And they can look at things and say, “well next time I’m going to do this” or “I’m going to try this differently”* (Teacher 1, Time 1). However, not all teachers were convinced that students at the Kindergarten level were capable. A few indicated that they wanted to give it more thought.

#### Theme 2: Barriers to developing metacognition

All participants were forthcoming with identifying ways in which they struggled with implementing metacognitive practices or encouraging the development of metacognition. These barriers included common classroom ones such as large classroom sizes which were grouped into the subtheme of competing demands. When one teacher was asked about any barriers to encouraging metacognition, one simply said *“Developmental readiness to a degree”* (Teacher 1, Time 1). One barrier related to developmental readiness was being too reactive as described by this ECE:

In our classroom I feel that many of our children are not aware of their thought processes as they are often reactive to situations around them and don’t stop to think things through to understand the why and how (ECE4, Time 2).

Some educators were quick to admit that the development of metacognition was often overlooked by them and other Kindergarten educators. This teacher explained further *“Often overlooked by educators, ‘not enough time,’ ‘difficult to mark/assess,’ ‘not as important as the hard skills”’* (Teacher 7, Time 2). This quotation emphasizes competing priorities and potentially a devaluing of metacognition for some educators. Another teacher admitted that metacognition was not a priority as described below.

I’m worried about kids falling through the cracks. So as much as I want you to become self-reflective and metacognitive about all of those things. It’s just one of those things. There’s only so many hours in a day, I just feel like I don’t get to it for some reason (Teacher 1, Time 1).

A different barrier was a lack of play time and having too much structured learning time. This ECE explained further *“I feel the structured learning environment and limited play-based learning in our classroom leaves little time”* (ECE 2, Time 1). However, it was promising to observe more effort toward the development of metacognition during the second time period including through the use of think-aloud protocols, ongoing dialog, and prompting (Time 1 and 2, Classroom Observation, Teacher 1, 4, and 7; ECE 1, 3, and 4).

#### Theme 3: Operationalization of strategies to facilitate metacognitive development

Despite reporting struggling with developing metacognition in their students, most educators were able to provide examples of practices that they implemented to promote the development of metacognition. Sometimes these strategies were described in relation to a specific context such as math with one educator explaining how they encouraged students to verbalize their strategies during number talks (Classroom Observation, Teacher 1, Time 1) or about literacy where a different educator discussed sharing book predictions. Educators tended to emphasize the need for explicit modeling of strategies through thinking out loud. This teacher explained, “…*if we don’t show that thinking part out loud or talking out loud for them, they’re not going to develop those skills”* (Teacher 8, Time 2). A different teacher consistently asked students to explain their thinking when they provided an answer (Classroom Observation, Teacher 2, Time 1). One teacher explained that *“Children are learning how best to complete tasks”* (Teacher 5, Time 2) in her classroom. This teacher shared examples observed during play:

I sometimes see that in terms of children actually having little out loud conversations with themselves. They’ll be building a tower of blocks and how can I put this one? I’ve got this, It fell off and they could try the same thing again. It fell off and they try the same thing and it fell off. And sometimes I hear them going, oh, this one’s got a curvy edge. It’s falling off. I’m going to try this one. So sometimes I actually hear kids reasoning through those things themselves, or I will hear things. They’re building something and they’ve got a car in a little ramp. Wow, this ramp is higher, that car went faster (Teacher 1, Time 2).

Other educators reiterated the role that play-based learning can have when working to develop student metacognition. One teacher reported that she did not have as much time as she would have liked to encourage the development of metacognition. She explained further:

It is a goal that I had hoped to reach more this year. I am working with a new ECE and needed this year to build that relationship. Hopefully, we will be able to put a system into place for next year where we can target specific children on certain days of the week to create time and space for [focusing] on deep reflective practice (Teacher 3, Time 2).

Another key subtheme centered around providing feedback after students had the opportunity to try strategies independently. Sometimes this feedback was provided by the educators while other times it was shared by peers. This teacher shared an example of providing more informal feedback during a conversation:

And I have conversations sort of informally with children about pieces of that. So for example, writing their name and “I notice that you’ve done this, you’re using all capital letters that’s shouting. We need to keep working on this. Get your name card.” And so having those conversations with kids (Teacher 1, Time 1).

Another mechanism for providing feedback included co-constructing with students as explained by this teacher:

…we take for granted that we can process everything in our head and then really quickly and have an idea. So I think for us, we tried to do everything with the kids [and] develop a learning chart with the kids (Teacher 8, Time 2).

KWL charts were also used by one teacher and ECE pair to help support metacognition. More specifically, the teacher and ECE would ask students to identify what they knew about a topic and write it down. They would then write down what the students wanted to learn. After completing the lesson, the educator would document what the students learned (Classroom Observation, Teacher 6 and ECE 5, Time 1). Finally, educators also provided students with the necessary vocabulary when sharing feedback. This teacher elaborated “…*so in Kindergarten I think giving them the language and explaining sort of terms and things, you know, vocabulary”* (Teacher 4, Time 2). Educators described how they used prompting through questioning to promote the development of metacognition as shared by this ECE “…*what were you thinking about that? you know, asking those questions of you know, well, why do you think the Caterpillar did this?”* (ECE1, Time 2). Another form of prompting was through the use of symbols to help students progress through steps. Many participants also emphasized the importance of a growth mindset and encouraging students to make mistakes. Despite having an incomplete understanding of metacognition, early years educators were trying a variety of different strategies to help promote metacognitive thinking within their Kindergarten classrooms.

### Research question (2) how do young children’s metacognition and self-regulatory behaviors evolve across two time periods as measured by educators?

The demographic variables explored were sex (male or female) and grade level (SK or SK). A complete overview of descriptive findings is available in Supplementary Appendix B. At Time 1, all means across items were higher for female students than male students when rated by the teachers except for one item. The one item was a motivational one where male students (*M* = 1.74) enjoyed solving problems more than female students (*M* = 1.71). Similarly, there was one motivational item at Time 2 where male students were rated more highly than female students by teachers. More specifically, male students (*M* = 2.18) initiated activities more than female students (*M* = 2.15). At Time 1, all means across items were higher for SK than JK students. This was also the case for Time 2 for teacher ratings.

At Time 1, all means across items were higher for female students than male students when rated by the ECEs. However, when looking at grade level, there were three prosocial items where junior kindergarten (JK) students had higher means than those in SK. More specifically, JK students (*M* = 1.60) were better able to resolve social problems with peers than those in SK (*M* = 1.5). For another prosocial item, JK students (*M* = 2.40) were better able to engage in independent cooperative activities with peers than their SK colleagues (*M* = 2.36). Finally, JK students (*M* = 2.08) were more aware of the feelings of others and helped and comforted others than their SK peers (*M* = 2.07). Similarly, at Time 2, all means across items were higher for female students than male students when rated by the ECEs. There was only one item at Time 2 where JK students had a higher mean than SK students according to the ECEs. The item was again a prosocial one whereby JK students (*M* = 1.56) were better able to resolve social problems with peers than SK students (*M* = 1.54).

Surprisingly, teacher ratings using the CHILD did not change significantly from Time 1 (*M* = 1.88, SD = 0.744) to Time 2 (*M* = 1.85, SD = 0.66), *t* (72) = 0.72, *p* > 0.05. Furthermore, teacher ratings decreased slightly from Time 2 to Time 1. A similar trend was observed for ECE ratings using the CHILD as they did not change significantly from Time 1 (*M* = 1.89, SD = 0.70) to Time 2 (*M* = 1.80, SD = 0.79), *t* (52) = 1.36, *p* > 0.05. ECE ratings also decreased slightly from Time 2 to Time 1.

## Discussion

This mixed methods study aimed to explore the development of metacognition in eight Kindergarten classrooms. A combination of classroom observations, interviews, and educator ratings provided insight into metacognitive behaviors in young children. When examining conceptions of metacognition, it was clear that both teachers and ECEs in the current study had a developing understanding of metacognition. They were able to provide the literal translation and the odd time articulate different components such as reflective thinking or planning. Furthermore, MEs were not mentioned at all by these educators. They tended to focus more on the awareness component. This is concerning when considering the model of metacognition described earlier given that MEs are key across the three levels. More specifically, it is proposed that MEs may activate the regulatory loop (Efklides, 2008). Without this activation, metacognition is not being developed to its fullest capacity in these young children.

The lack of a deeper conceptual understanding may not be surprising as previous studies have found similar results. For example, one study examining the connection between assessment and metacognition at the elementary level reported teachers conceptualizing metacognition as the ability to understand one’s thinking (Braund and DeLuca, 2018). They also noted a similar gap with little to no mention of MEs (Braund and DeLuca, 2018). A different study examining assessment in Kindergarten classrooms demonstrated that only 5 of 20 teachers mentioned metacognition in relation to SR and defined it as understanding an individual’s learning (DeLuca et al., 2020).

Additionally, the Kindergarten curriculum in Ontario is centered around SR with some consideration for metacognition. A recent document analysis of this curriculum document was conducted to better understand how SR was operationalized throughout the document. One of their conclusions was the need to move toward a more holistic conceptualization of metacognition with the inclusion of all three components (metacognitive regulation, metacognitive awareness, and ME). This was identified as a need given that the policy document focused on the use of language to share one’s learning without considering planning, monitoring, or evaluating behaviors (Braund and Timmons, 2021). Given the focus of the Kindergarten curriculum document, it makes sense why the educators in this study emphasized sharing one’s thinking and providing students with the necessary vocabulary to describe their thinking. One educational implication is that we need to provide in-service teachers with additional programming around the different facets of metacognition to help with the development of a more fulsome conceptualization. Additionally, this should begin in pre-service programming so that new teachers entering the system have stronger conceptions of metacognition.

Educators in the current study also reported a few key barriers that impacted their ability to integrate metacognition into their classrooms. These barriers included competing demands and the developmental readiness of students. It is well understood that teachers have a lot that they need to accomplish in the classroom including the development of SR, facilitating learning, and assessing learners. Even with two adults in the room (a teacher and ECE), the Kindergarten classroom remains chaotic with young learners. A few educators in the current study also reiterated the role that developmental readiness plays. This is interesting given that previous research has demonstrated that children are capable of metacognitive and self-regulatory behaviors (Blair and Razza, 2007; Dignath et al., 2008; Whitebread et al., 2010; Erdmann and Hertel, 2019; Perry, 2019). It may be that educators lack many examples of concrete practices aimed to develop metacognition. Furthermore, some practices may not be developmentally appropriate for learners in Kindergarten. Therefore, educators need to be exposed to concrete practices that are appropriate for Kindergarten students. This includes providing educators with concrete examples of how MEs can be developed in the Kindergarten classroom for more substantiative metacognitive development. Educators in the current study used prompting and questioning as one technique to promote the development of metacognition. This is in alignment with another study that explored the use of metacognitive questions across three schools in the early years during literacy lessons. The questions were largely phrased around “how” and “why” encouraging students to explain their thinking. However, in the lessons that were analyzed, these types of questions comprised 5–15% of the total number of questions analyzed (Gourlay et al., 2020). This low frequency suggests that metacognitive questions could be used to a greater extent in early years classrooms and across subjects.

Other strategies used in the current study included the provision of feedback related to metacognition. Multiple educators explained that feedback was ongoing throughout the day and that they would provide students with the opportunity to try strategies independently before providing them with feedback. One study examined the impact of providing different types of feedback on student learning in grade 5 classrooms. Corrective feedback helped students to work toward their immediate goals; however, metacognitive feedback better prepared students for other learning activities even after metacognitive support was removed. However, the authors clearly emphasized the need for students to receive explicit instruction on metacognitive strategies and be allowed to practice using the strategies. Otherwise, the metacognitive feedback may not help novice learners who had little prior knowledge (Tan et al., 2006).

Related to educator ratings, generally, female students outperformed male students across the metacognition and SR items in the CHILD. However, in the few instances where male students were rated more highly for SR and metacognition behaviors, they were for motivational items. One study found significant differences according to gender in Grade 8 students. The differences were observed for specific metacognition items such as “My performance depends on my will and my effort” and “I know what teachers expect me to learn” (Liliana and Lavinia, 2011). The first item may involve motivational processes and thus aligns with the current study that found differences among male students even at the Kindergarten level, although the differences in the current study were descriptive trends rather than significant differences.

Similar trends were observed as to how teachers and ECEs rated students using the CHILD. This concordance of ratings helps to provide additional validity evidence for the use of the measure in different educator groupings. Surprisingly, the teacher and ECE ratings did not differ significantly at Time 2 when compared to Time 1 for the current study. The study was originally designed to capture changes in SR and assessment practices across the 12 weeks. Yet, there is a chance that 12 weeks were not enough to capture changes in these students’ behaviors. It may be posited that a change in metacognition and SR behaviors could be observed if Time 1 was in September and Time 2 was closer to the end of the school year such as in May. There may also be events in the lives of students for the current study that may have caused them to struggle with regulation at Time 2 when compared to Time 1.

One mixed methods study compared metacognitive beliefs and practices for pre-service (*n* = 43) and in-service (*n* = 45) teachers. Both groups of teachers recognized the importance of metacognition however, in-service teachers reported greater integration than pre-service teachers. In-service teachers tended to report more concrete strategies such as explicit instruction, use of think-aloud protocols, and students as active agents, whereas pre-service teachers tended to be more idealistic without concrete examples from their practice. Additionally, in-service teachers tended to have deeper conceptualizations of metacognition making connections to higher order thinking and the use of metacognitive strategies across domains (Braund and Soleas, 2019). These findings reiterate the importance of not just teaching about metacognition but also how to integrate metacognition across grade levels. Educators seem to agree on the importance of metacognition but continue to struggle with its implementation. Kindergarten is of particular interest because it sets the foundation for later learning. It may be that Kindergarten teachers need examples of how metacognition practices could be adapted for use in their classrooms. It was clear in the current study that all educators wanted to integrate more metacognition practices in their classrooms but struggled with competing demands. Perhaps if they were provided with examples of how these strategies could be applied across subject domains it may help with their integration.

### Limitations

This mixed methods study had several limitations including that all data were collected from one school board. There was diversity across schools and students. Additionally, only six of the eligible eight ECEs participated in the study. Therefore, some of their perspectives may not have been captured relating to the development of metacognition. Specific to the measurement, the CHILD measure was completed by educators who may have interpreted the items differently without having more formal training. This emphasizes the need for triangulating across measures for metacognition in children.

### Future research

Future research should add additional timepoints including measuring metacognition and SR behaviors at the beginning of the year to capture a baseline. More studies need to take a longitudinal approach in the early years focused on understanding the development of metacognition as children develop. Given that the gender and sex differences were observed for select items, future work should continue to explore metacognitive behaviors according to gender or sex. Measures need to be triangulated to ensure an accurate demonstration of metacognition such as through the use of think-aloud protocols, classroom observations, interviews, and self-report items. Additionally, as we understand more about how metacognition is operationalized in the early years, interventions can be designed and implemented to facilitate the development in Kindergarten.

## Conclusion

This research highlights empirical practices that Kindergarten educators can use to help facilitate metacognitive thinking. Despite having barriers impacting their implementation, educators in the current study worked to encourage metacognitive thinking using developmentally appropriate strategies. Furthermore, findings highlight a need to better support Kindergarten educators with developing metacognitive thinking in their students through explicit examples of strategies for use across subject domains and substantiating their conceptualization.

## Data availability statement

The datasets presented in this article are not readily available because additional ethics approval would be required to share raw data beyond the research team specified in the original ethics application. Requests to access the datasets should be directed to HB, hlab@queensu.ca.

## Ethics statement

The studies involving human participants were reviewed and approved by General Research Ethics Board at Queen’s University. Written informed consent to participate in this study was provided by the participants or their legal guardian/next of kin.

## Author contributions

HB lead the study design with input from her dissertation committee, collected and analyzed all data, wrote this manuscript herself, and is accountable for the accuracy and integrity of the work.
