# Supplementary material for: Thinking about Kindergarten thinking: A mixed methods study
Source: Front Psychol. 2022 Sep 2;13:933541. doi: 10.3389/fpsyg.2022.933541 (PMC9481266; doi:10.3389/fpsyg.2022.933541)
Supplement: Supplementary file 1 [file Table_1.DOCX]

Supplementary Material

# Supplementary Data

Supplementary Material should be uploaded separately on submission. Please include any supplementary data, figures and/or tables. All supplementary files are deposited to FigShare for permanent storage and receive a DOI.

Supplementary material is not typeset so please ensure that all information is clearly presented, the appropriate caption is included in the file and not in the manuscript, and that the style conforms to the rest of the article. To avoid discrepancies between the published article and the supplementary material, please do not add the title, author list, affiliations or correspondence in the supplementary files.

# Supplementary Figures and Tables

**Appendix A**

**Additional Quotations**

| **Theme** | **Subthemes** | **Codes** | **Quotations** |
| --- | --- | --- | --- |
| 1. Conceptualization of metacognition | Surface level understanding  Multifaceted  Capacity | Thinking about thinking  Awareness of processing  Awareness of thinking  Reflective thinking    Planning  K students are capable  Unsure if students are capable | *“metacognition, thinking about your thinking?”* (Teacher 4, Time 2).  *“Well I think like metacognition, is that not being aware of… how you process?”* (Teacher 6, Time 2).  *“…is that not being aware of how you think?”* (Teacher 6, Time 2).  *“It is a reflective piece, can include reflecting on the process of a task/learning”* (Teacher 7, Time 2).  *“Planning how to approach a task”* (ECE 2, Time 2).  *“I think a lot of people think kindergarteners can’t do this. I think they can”* (Teacher 4, Time 2).  *“They’re just learning about learning at this point. So they don’t, it’s tricky because they don’t know themselves well enough to know what they do and don’t know”* (Teacher 1, Time 2). |
| 1. Barriers to developing metacognition | Competing demands  Developmental readiness | Large class sizes  Different developmental trajectories | *“Partly the trickiness in kids being really young but the other piece is just time on my part and feeling like I’m torn with that many kids in the class”* (Teacher 1, Time 1).  *“And they're all at different stages"* (ECE1, Time 1). |
| 1. Operationalization of strategies to facilitate metacognitive development | Thinking out loud  Providing feedback  Prompting | Reasoning out loud  Sharing thinking  Feedback about strategies  Asking questions to prompt thinking  Symbols to help progress through steps | *“But it's getting kids to talk about, there's an arrangement of dots, there's five, how do they explain to me that there were five? So they're able to say, well, I counted, I did this, I put three on this finger, I put two on this finger. I saw in a group these and I kept counting on. So it's that explanation of strategies that they're, that they're using”* (Teacher 1, Time 2).  *“I think that for us in this room it’s just kind of always talking, showing the kids your thinking. So…thinking out loud for them so that they can learn how to develop those thinking skills”* (Teacher 8, Time 2).  *“or try to resolve issues themselves. Students can try strategies independently and they will get social feedback almost immediately”* (Teacher 7, Time 2).  *“And I think that’s something that’s just starting probably in kindergarten and we’re building on and maybe asking questions to prompt them to think about things where they might not have on their own”* (Teacher 2, Time 2).  *“Using fixed symbols helps them work through those steps”* (Teacher 2, Time 2). |

**Appendix B-Descriptive Findings**

**Table 1:** CHILD items for Time 1. ‘T’ represents teacher and ‘ECE’ represents early childhood educator. JK represents Junior Kindergarten whereas SK represents Senior Kindergarten.

| Demographics | **10. CHILD T1: ProSocial: Is aware of feelings of others and helps and comforts** | | **11. CHILD T1: Cognitive: Is aware of own strengths and weaknesses** | | **12. CHILD T1: Cognitive: Can speak about how they have done something or what they have learnt** | | **13. CHILD T1: Cognitive: Can speak about future planned activities** | | | | **14. CHILD T1: Cognitive: Can make reasoned choices and decisions** | | **15. CHILD T1: Cognitive: Asks questions and suggests answers** | | **16. CHILD T1: Cognitive: Uses previously taught strategies** | | **17. CHILD T1: Cognitive: Adopts previously heard language for own purposes** | | | | **18. CHILD T1: Motivational: Finds own resources without adult help** | | |
| --- | --- | --- | --- | --- | --- | --- | --- | --- | --- | --- | --- | --- | --- | --- | --- | --- | --- | --- | --- | --- | --- | --- | --- |
| **Educator** | T | ECE | T | ECE | T | ECE | T | | ECE | | T | ECE | T | ECE | T | ECE | T | | ECE | | T | | ECE |
| **Female** | 2.33 | 2.38 | 1.90 | 1.87 | 2.14 | 2.28 | 2.23 | | 2.24 | | 2.23 | 2.38 | 2.18 | 2.24 | 2.05 | 2.17 | 2.05 | | 1.97 | | 1.97 | | 2.31 |
| **Male** | 1.56 | 1.71 | 1.28 | 1.25 | 1.72 | 1.75 | 1.72 | 1.62 | | 1.74 | | 1.88 | 1.72 | 1.83 | 1.62 | 1.71 | 1.59 | 1.46 | | 1.74 | | 1.94 | |
| **JK** | 1.82 | 2.08 | 1.36 | 1.26 | 1.58 | 1.68 | 1.62 | 1.52 | | 1.72 | | 1.92 | 1.54 | 1.80 | 1.56 | 1.60 | 1.51 | 1.28 | | 1.74 | | 1.90 | |
| **SK** | 2.08 | 2.07 | 1.82 | 1.89 | 2.28 | 2.36 | 2.33 | 2.36 | | 2.26 | | 2.36 | 2.36 | 2.29 | 2.10 | 2.29 | 2.13 | 2.14 | | 1.97 | | 2.36 | |

| Demographics | **19. CHILD T1: Motivational: Develops own ways of carrying out tasks** | | **20. CHILD T1: Motivational: Initiates activities** | | **21. CHILD T1: Motivational: Plans own tasks, targets, and goals** | | **22. CHILD T1: Motivational: Enjoys solving problems** | | |
| --- | --- | --- | --- | --- | --- | --- | --- | --- | --- |
| **Educator** | T | ECE | T | ECE | T | ECE | T | ECE |  |
| **Female** | 2.03 | 2.07 | 2.08 | 2.10 | 1.87 | 1.74 | 1.71 | 1.91 |  |
| **Male** | 1.77 | 1.67 | 1.95 | 1.71 | 1.67 | 1.48 | 1.74 | 1.61 |  |
| **JK** | 1.74 | 1.48 | 1.82 | 1.60 | 1.46 | 1.18 | 1.49 | 1.44 |  |
| **SK** | 2.05 | 2.25 | 2.21 | 2.21 | 2.08 | 2.02 | 1.97 | 2.07 |  |

| **Demographics** | **1. CHILD T2: Emotional: Can speak about own behaviour and consequences** | | **2. CHILD T2: Emotional: Tackles new tasks confidently** | | **3. CHILD T2: Emotional: Can control attention and resist distraction** | | **4. CHILD T2: Emotional: Monitors progress and seeks help appropriately** | | **5. CHILD T2: Emotional: Persists in the face of difficulties** | | **6. CHILD T2: Prosocial: Negotiates when and how to carry out tasks** | | **7. CHILD T2: ProSocial: Can resolve social problems with peers** | | **8. CHILD T2: ProSocial: Shares and takes turns independently** | | **9. CHILD T2: ProSocial: Engages in independent cooperative activities with peers** | |
| --- | --- | --- | --- | --- | --- | --- | --- | --- | --- | --- | --- | --- | --- | --- | --- | --- | --- | --- |
| **Educator** | T | ECE | T | ECE | T | ECE | T | ECE | T | ECE | T | ECE | T | ECE | T | ECE | T | ECE |
| **Female** | 2.15 | 2.50 | 1.96 | 1.82 | 1.85 | 2.18 | 2.01 | 2.21 | 1.86 | 1.75 | 2.09 | 1.86 | 1.90 | 1.68 | 2.16 | 2.39 | 2.29 | 2.63 |
| **Male** | 1.64 | 1.92 | 1.59 | 1.44 | 1.56 | 1.52 | 1.62 | 1.60 | 1.44 | 1.16 | 1.74 | 1.32 | 1.51 | 1.40 | 1.87 | 1.83 | 1.99 | 2.08 |
| **JK** | 1.55 | 2.04 | 1.55 | 1.40 | 1.51 | 1.72 | 1.62 | 1.84 | 1.47 | 1.28 | 1.69 | 1.20 | 1.50 | 1.56 | 1.85 | 2.08 | 2.00 | 2.24 |
| **SK** | 2.23 | 2.39 | 1.97 | 1.86 | 1.89 | 2.00 | 1.99 | 2.00 | 1.81 | 1.64 | 2.12 | 1.96 | 1.91 | 1.54 | 2.16 | 2.19 | 2.26 | 2.48 |

**Table 2:** CHILD items for Time 2. ‘T’ represents teacher and ‘ECE’ represents early childhood educator. JK represents Junior Kindergarten whereas SK represents Senior Kindergarten.

| Demographics | **10. CHILD T2: ProSocial: Is aware of feelings of others and helps and comforts** | | **11. CHILD T2: Cognitive: Is aware of own strengths and weaknesses** | | **12. CHILD T2: Cognitive: Can speak about how they have done something or what they have learnt** | | **13. CHILD T2: Cognitive: Can speak about future planned activities** | | **14. CHILD T2: Cognitive: Can make reasoned choices and decisions** | | **15. CHILD T2: Cognitive: Asks questions and suggests answers** | | **16. CHILD T2: Cognitive: Uses previously taught strategies** | | **17. CHILD T2: Cognitive: Adopts previously heard language for own purposes** | | **18. CHILD T2: Motivational: Finds own resources without adult help** | |
| --- | --- | --- | --- | --- | --- | --- | --- | --- | --- | --- | --- | --- | --- | --- | --- | --- | --- | --- |
| **Educator** | T | ECE | T | ECE | T | ECE | T | ECE | T | ECE | T | ECE | T | ECE | T | ECE | T | ECE |
| **Female** | 2.14 | 2.50 | 2.01 | 2.04 | 2.14 | 2.32 | 2.15 | 2.25 | 2.19 | 2.29 | 2.19 | 2.14 | 2.17 | 2.21 | 2.06 | 2.18 | 2.01 | 2.18 |
| **Male** | 1.73 | 1.84 | 1.49 | 1.24 | 1.60 | 1.64 | 1.80 | 1.56 | 1.76 | 1.64 | 1.68 | 1.68 | 1.70 | 1.60 | 1.66 | 1.32 | 1.97 | 1.72 |
| **JK** | 1.83 | 2.16 | 1.46 | 1.32 | 1.59 | 1.64 | 1.63 | 1.64 | 1.73 | 1.76 | 1.57 | 1.64 | 1.57 | 1.60 | 1.57 | 1.56 | 1.86 | 1.88 |
| **SK** | 2.03 | 2.21 | 2.03 | 1.96 | 2.13 | 2.32 | 2.31 | 2.18 | 2.22 | 2.18 | 2.30 | 2.18 | 2.28 | 2.21 | 2.15 | 1.96 | 2.12 | 2.04 |

| Demographics | **19. CHILD T2: Motivational: Develops own ways of carrying out tasks** | | **20. CHILD T2: Motivational: Initiates activities** | | **21. CHILD T2: Motivational: Plans own tasks, targets, and goals** | | **22. CHILD T2: Motivational: Enjoys solving problems** | | |
| --- | --- | --- | --- | --- | --- | --- | --- | --- | --- |
| **Educator** | T | ECE | T | ECE | T | ECE | T | ECE |  |
| **Female** | 1.97 | 1.93 | 2.15 | 2.32 | 2.00 | 2.07 | 2.00 | 1.93 |  |
| **Male** | 1.97 | 1.60 | 2.18 | 1.68 | 1.41 | 1.44 | 1.64 | 1.60 |  |
| **JK** | 1.81 | 1.40 | 2.00 | 1.72 | 1.47 | 1.48 | 1.58 | 1.64 |  |
| **SK** | 2.14 | 2.11 | 2.34 | 2.29 | 1.91 | 2.04 | 2.04 | 1.89 |  |
